# Volatile Composition of Fortification Grape Spirit and Port Wine: Where Do We Stand?

**DOI:** 10.3390/foods12122432

**Published:** 2023-06-20

**Authors:** Sónia Gomes Ribeiro, Cátia Martins, Tiago Tavares, Alisa Rudnitskaya, Fernando Alves, Sílvia M. Rocha

**Affiliations:** 1 Department of Chemistry & LAQV-REQUIMTE, University of Aveiro, Campus Universitário de Santiago, 3810-193 Aveiro, Portugal; srgr@ua.pt (S.G.R.); catiamartins@ua.pt (C.M.); tiago96@ua.pt (T.T.); 2 Department of Chemistry & CESAM, University of Aveiro, Campus Universitário de Santiago, 3810-193 Aveiro, Portugal; alisa@ua.pt; 3 Symington Family Estates, Vinhos S.A. Travessa Barão de Forrester, 86, 4400-034 Vila Nova de Gaia, Portugal; fernando.alves@symington.com

**Keywords:** grape spirit, Port wine, fortification, volatile components, aroma, gas chromatography

## Abstract

Port wine’s prominence worldwide is unequivocal and the grape spirit, which comprises roughly one fifth of the total volume of this fortified wine, is also a contributor to the recognized quality of this beverage. Nonetheless, information about the influence of the grape spirit on the final aroma of Port wine, as well as its volatile composition, is extremely limited. Moreover, the aroma characteristics of Port wines are modulated mainly by their volatile profiles. Hence, this review presents a detailed overview of the volatile composition of the fortification spirit and Port wine, along with the methodologies employed for their characterization. Moreover, it gives a general overview of the Douro Demarcated Region (Portugal) and the relevance of fortification spirit to the production of Port wine. As far as we know, this review contains the most extensive database on the volatile composition of grape spirit and Port wine, corresponding to 23 and 208 compounds, respectively. To conclude, the global outlook and future challenges are addressed, with the position of the analytical coverage of the chemical data on volatile components discussed as crucial for the innovation centered on consumer preferences.

## 1. Introduction

Port wine is a fortified wine exclusively produced in the Douro Demarcated Region (Portugal) under very specific conditions resulting from natural and human factors. Recognized with protected designation of origin, Port wine is an internationally appreciated fortified wine, and in 2020, represented ca. 56% of the Douro Demarcated Region’s annual wine production, which also includes Douro wines (red, white, and *rosé*), Moscatel and Sparkling wines. Port wine accounts for approximately 66% of the total income generated by Portugal in wine exports, corresponding to a value of EUR 339 million and volume of about 68 million liters [1].

Port wine aroma characteristics are modulated by a network of factors, such as the terroir (including microbiome), varieties, yeast metabolites and winemaking procedures that include a wide set of steps, namely the fortification with grape spirit (ca. 77% *v*/*v* ethanol) and wood ageing [2,3]. The fermentation stoppage is obtained by adding a grape spirit (also named *aguardente*) to achieve a final alcoholic content of around 19% (*v*/*v*) [4,5]. As the grape spirit comprises roughly one fifth of the total volume of this fortified wine, it is a potential contributor to the global quality of this beverage, including the aroma notes. Nonetheless, the information about the influence of the grape spirit on the final aroma of Port wine, as well as the grape spirit volatile composition, is extremely limited. Grape spirit volatile organic compounds (VOC) arise from a network of variables, namely from original grape metabolism, yeast metabolism, and fermentation, among others, and may suffer modifications during distillation.

**Figure 1:**
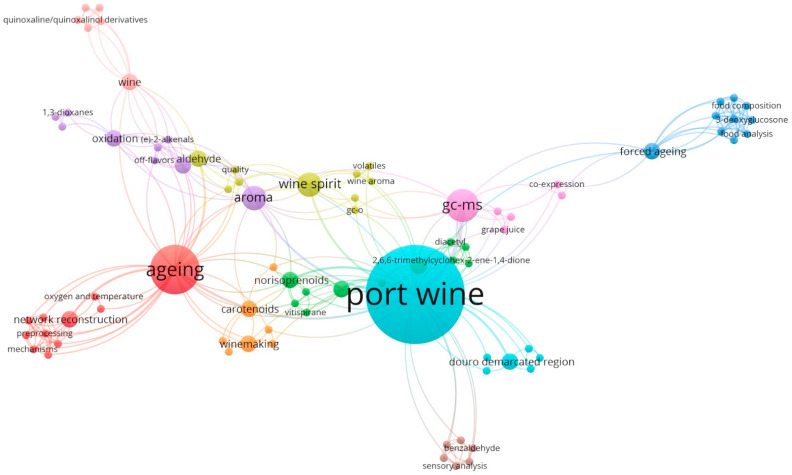
Analysis of the co-occurrence links between keywords, obtained using the software VOSviewer 1.6.16, considering the bibliographic information available in the Scopus database concerning the aroma/volatile composition of Port wines and fortification grape spirit (also reported in the literature as wine spirit and *aguardente*) [3,5,6,7,8,9,10,11,13,14,15,16,17,18,19,20,21,22,23,24,25,26,27,28,29].

In order to systematize the data available in the literature about the volatile composition of grape spirits (used in the fortification step) and Port wine, a search in the Scopus database was performed using the following combinations of keywords: “Port wine AND aroma”; “Port wine AND volatile compounds” and “Port wine AND wine spirit” [3,5,6,7,8,9,10,11,12,13,14,15,16,17,18,19,20,21,22,23,24,25,26,27,28,29]. This bibliographic search led to the construction of the diagram presented in Figure 1, which allows the visualization of the bibliometric networks between the retrieved results from Scopus, in this case, using the co-occurrence links between keywords. The circles and the label size, along with their relatedness (proximity), were determined according to the number of citations and sources in which the keywords occurred simultaneously (Figure 1). This created bibliometric network allowed us to observe that the most prominent keywords and closest ones were Port wine, followed by ageing, GC-MS (gas chromatography–mass spectrometry), wine spirit (also reported as grape spirit and *aguardente*), and aroma. It was also possible to observe that the wine spirit is directly connected to wine aroma, volatiles, quality, and aldehyde content. It was also interesting to see the proximity of the keywords quality and off-flavor, which were closest (and possibly related) to oxidation, aldehydes and (E)-2-alkenals. Other keywords with lower bibliographic representativeness, but still with some impacts, were: Douro Demarcated Region, wine making, forced aging, carotenoids, and norisoprenoids.

This search also shedded light on the fact that the available information about the fortification spirit used in the production of Port wine is relatively scarce, as well as the knowledge regarding the volatile chemical composition of fortified wine. Additionally, few studies have been performed on the impact of grape spirit composition on the color, taste and aroma profile of Port wine [3,29]. A chemesthetic mouth-warming feeling provided by the fortification spirit, as well as the high sugar content of Port wine (resulting from the arrest of the fermentation by the addition of fortification spirit), allows a reduction of tannins’ intensity that consequently lowers wines’ astringency [5]. In terms of aroma, the grape spirit has been empirically considered as neutral by producers, introducing a higher ethanol amount to Port wine. However, previous research has shown that grape spirits contain volatile organic compounds potentially impactful for the aroma bouquet of Port wine, namely ethyl esters (ethyl hexanoate, ethyl octanoate, ethyl decanoate, and ethyl hydrocinnamate), monoterpenic compounds (α-terpineol and linalool) and benzaldehyde [3]. Hence, this review aims at in-depth mapping of the volatile profiles of grape spirit, used for the fortification of Port wine, and of Port wine, and the methodologies that were used for these characterizations. Firstly, the characteristics of the Douro Demarcated Region and the uniqueness of Port wine’s production will be briefly presented, focusing on the fortification step. Finally, the volatile composition of the spirits and Port wines will be covered, and, as far as possible taking into account the limited literature available, they will be discussed in an integrated way.

## 2. Douro Demarcated Region Characteristics

**Figure 2:**
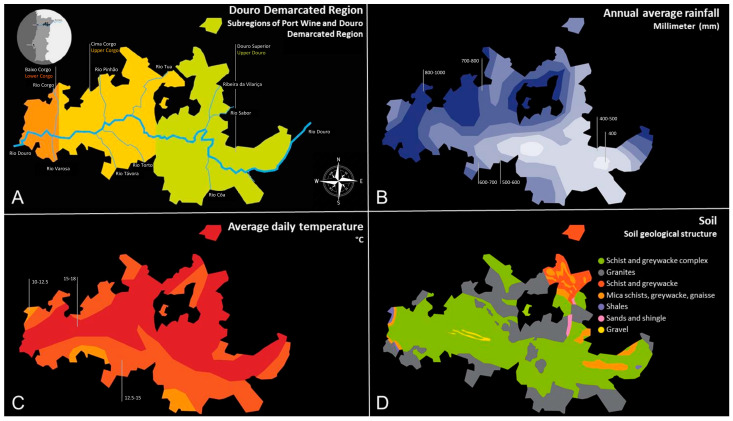
Douro Demarcated Region (DDR), adapted from Museu do Douro [31]: (**A**) Douro Demarcated Region divided into three subregions (orange—Baixo Corgo, yellow—Cima Corgo, green—Douro Superior) and the rivers present along this area, including the main one, the Douro River that crosses all of the DDR; (**B**) annual average rainfall in millimeters (mm), rainfall decreases in the direction of Spanish border; (**C**) average daily temperature, which is usually higher in the northern margin of the river; (**D**) soil geographic structure of Douro valley. ©2015, Museu do Douro.

Douro Demarcated Region (DDR, Figure 2) is the first demarcated and regulated region in the world, established in 1756. To ensure the superior quality of Port wine, in accordance with the Decree-Law No. 97 of 23 April 2012, the Instituto dos Vinhos do Douro e Porto (IVDP), founded in 1933, is the public body responsible for the certification, regulation of the production, and approval of the wines and the wine spirits produced in the Douro Demarcated Region. The IVDP guarantees the quality, authenticity, and the protection of the denomination of origin Douro and Port, by following specific criteria based on the soil, climate, and viticultural parameters [30].

Douro Demarcated Region extends over a total area of about 250,000 ha, with 43,167 ha of vineyards, of which 33,118 ha are intended for Port wine production, located on the steep hillsides along the course of the Douro River [5]. It is divided into three distinct subregions: Baixo Corgo, Alto Corgo, and Douro Superior (Figure 2A).

The subregions of DDR are delimitated according to their edaphoclimatic conditions and socio-economic factors. Baixo Corgo is part of the oldest viticultural area also known as *Alto-Douro Vinhateiro*, presenting the largest proportion covered with vines. The Baixo Corgo is associated with the most distinctive Port wines, and it is where the majority of the largest and most renowned *quintas* (estates) are located. Douro Superior is the largest and the most recent expansion of the region, being the closest to the Spanish border and incorporated only in the 20th century [7]. In terms of annual precipitation volume (Figure 2B), the rainfall decreases in the direction of the Spanish border, with the values varying between 400 mm (Douro Superior) and 1000 mm (Baixo Corgo). Sun exposure is a physiographic factor of great importance for the climatic characterization of any region, particularly the Douro Demarcated Region, since it provides a better understanding of the behavior of the vines in distinct situations. The northern margin of the river is under the influence of dry southerly winds, while the southern margin is exposed to the colder and wetter northerly winds and less sunshine. The air temperature is higher at the northern margin compared to the southern one (Figure 2C). The average annual temperatures range from 11.8 to 16.5 °C. The diurnal and annual temperatures are higher in Douro Superior compared to the Baixo Corgo, which can be explained by the distance from the sea [1,32]. In terms of altitude, the region presents an average elevation of 443 m, ranging from 40 m to just above the 1400 m [33]. Douro valley has a mountainous topography dominated by schistose-layered rock with outcrops of granite, with moderated to steep slopes and diverse exposures (Figure 2D) [34]. Overall, this region is characterized by low precipitation, high temperatures and high radiation exposure, which contributes to water and thermal stress, specifically in the Cima Corgo and Douro Superior sub-regions [33].

Wine is produced in a limited geographic and climatic area, which might put wine grapes at severe risk from climate change and fluctuation. The most crucial elements affecting winegrape development and yield are drought and temperature [35]. By promoting different ripening profiles and accelerated cultivar growth, climate variations and fluctuations can have an impact on wine quality. Therefore, in warmer environments, grapevines reach their sugar ripeness earlier, and flavors do not develop accordingly, resulting in unbalanced wines with higher alcohol content and lower acidity [34,35]. Projections indicate that further warming may range from 0.8–6.6 °C from 2020 to 2080, while precipitation during the growing season is projected to decline by up to 7–22% over the same period [35]. A shift toward warmer and drier conditions is foreseen in this region, which can impact the grape maturation and wine production [34]. DDR’s geographic location, orographic characteristics, surrounding flora, and grapevine biodiversity may mitigate the impact of climate change, helping to create meso- and microclimate conditions that can enable spatial adaptation strategies and provide alternatives for agricultural methods, diminishing the impact of climate change on DDR viticulture suitability [34,35].

A wide range of grape varieties are allowed for Port wine production. According to the Portuguese Official Gazette No. 243/2017, Ordinance No. 383/2017 and Portuguese Official Gazette No. 91/2001, Ordinance No. 413/2001, there are in total 115 approved cultivars in the Douro Appellation, of which 110 are authorized to produce Port wine [36,37], with the largest planted area occupied by Touriga Franca, Tinta Roriz, Tinta Barroca, Touriga Nacional and Tinta Amarela for red cultivars and Côdega, Malvasia Fina, Rabigato, Gouveio and Viosinho for white cultivars [38]. Due to its aroma complexity and the intense red coloration, Touriga Nacional, a traditional *Vitis vinifera* cultivar, is the finest and most notorious grape variety among all of the cultivars allowed for Port wine production. However, its low yield and sensitivity to physiological disorders, such as millerandage and coulure, may represent huge challenges [19].

## 3. Port Wine’s Production: The Relevance of the Fortification Step

**Figure 3:**
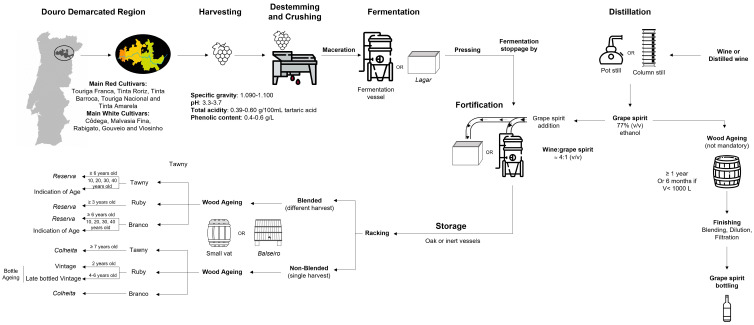
Schematic representation of Port wine production process, including fortification spirit production [19,41,42].

Port wine production has endured for centuries, and its vinification process respects the traditional methods but also takes advantages of new advances in viticulture and enology areas, such as the continuous advances in the development of new equipment, technologies, and enological adjuvants, among others. Figure 3 illustrates the main steps involved in Port wine’s production, which includes multiple and sequential steps, namely the fortification step with grape spirits (ca. 77% *v*/*v* ethanol).

The harvesting of the grapes and the winemaking process itself take place in the DDR, predominantly in regional cooperatives or *quintas*. But the subsequent steps, namely the maturation and bottling, occur mainly in Vila Nova de Gaia, a city located across from Porto, on the south side of the estuary of the Douro River. The Entreposto of Gaia was created in 1926 for wine storage, because, at the time, companies associated with the Port wine trade were obliged to move or build cellars within Gaia for the ageing of their wines. Since 1986, the exportation of wines directly from the DDR has been authorized [7,39].

All of the stages illustrated in Figure 3, from the collection of the grapes to the aging of the wine, are properly outlined according to the style of wine that is intended to be produced and are also adapted to the conditions of each vineyard, climatic conditions of the year and cellar facilities. Each producer imprints his own style, respecting Port wine regulations (Regulation No. 84/2010 of 8 February 2010) [40] and considering market trends and the current lifestyle. For instance, the selection of the grape varieties may be performed, according to their anthocyanin and tannins content, and it is essential to ensure the adequate preservation of red wine color and mouthfeel throughout the maturation step, which can take many years or even decades. Indeed, producers’ decision-making is increasingly based on objective information; for example, the harvesting process takes place when the grapes present a specific gravity between 1.090 and 1.100, a total acidity between 3.9 and 6.0 g/L (expressed as tartaric acid), a pH around 3.3 to 3.7 (in warmer regions the pH can be as high as 4.0) and phenolic compound concentrations between 0.4 and 0.6 g·L^−1^ [41].

It is not at all the purpose of this section to detail the various stages involved in the production of Port wine, for which proper regulations and publications already exist [5,7,19,41,42]; however, production of grape spirits and the fortification stage will be emphasized.

The fortification spirits used to produce wine likely to obtain the denomination of origin Porto and Moscatel do Douro must be previously certified by the IVDP, according to Regulation No. 84/2010 of 8 February 2010 [40]. The certification procedure depends on the fulfillment of requirements related to organoleptic properties such as clarity (limpid), color (colorless), aroma and taste (absence of any aroma extraneous to the raw material or defective), and physicochemical characteristics, which include clarity, color, alcoholic grade, total acidity, density, volatile compound content (for instance, ethyl carbamate, total higher alcohols, and acetaldehyde) and mineral content (namely calcium, copper, and iron) [40].

Distillation is the main technological process used to produce grape spirit and consists of the separation of ethanol and other flavor compounds from the water and their transfer to the distillate. There are two main types of distillation equipment. The more traditional method is batch distillation, with a pot still, which originates heavily flavored distillates. Basically, it consists in a copper vessel with a steam tube on top, where the alcohol vapor accumulates. This tube leads to a water-cooled condenser, which allows ethanol to condense. This type of distillation equipment is often used to produce fruit distillates, brandies, and whiskies. Continuous stills consist in column stills, usually up to three, in which the steam is separated by means of reflux of the descending liquid and is carried out to the following plates where it is again concentrated to the desired alcoholic percentage [43]. Considering the capacity of extracting a higher ethanol concentration, column stills are more cost-effective than pot stills, with the latter technique being more laborious, time consuming, and less efficient energetically [44]. Both types of distillers allow the separation of the different fractions, commonly referred to as “cuts”. Compounds with higher volatility, such as acetaldehyde, acetal and ethyl acetate, are present in the “head cut”. Conversely, the “tail cut” contains volatile compounds with higher boiling points, such as ethyl esters of long chain fatty acids. Both fractions include undesirable volatile compounds that can impart off-flavors to the final product; hence, it is essential to separate these compounds from the “heart cut”, which represents the fraction rich in aroma compounds relevant for the organoleptic quality of the spirit [43]. A fortification grape spirit should exhibit a balance of volatile components, to meet the requirement of not presenting any aroma extraneous to the raw material or defective [44]. The grape spirit may be used for the fortification or may be aged in wood barrels and marketed as aged brandy (Figure 3).

Fortification grape spirit is added to fermenting must after 2 to 3 days from the start of the fermentation process, when the sugar content is at least 17.5 g/L. Non-highly rectified grape spirit is added to achieve a final alcoholic content of around 19% (*v*/*v*), representing a distinctive and crucial step [4,5]. The grape spirit is added at a ratio of one part of grape spirit to four parts of fermenting must. Grape spirits’ addition results in fermentation stoppage by induction of toxicity in yeasts due to high alcohol content (ca. 77% *v*/*v* ethanol). This step also promotes anthocyanin and tannin solubilization and the precipitation of insoluble matter [5,19]. The addition of grape spirits is especially relevant for young Port wine aroma and flavor, as it induces a warm feeling in the mouth and reduces the intensity of tannins and astringency [5].

Port wines can be categorized according to their sweetness and color. According to the sugar content, Port wines are classified as extra dry, dry, semi-dry, sweet, or very sweet. A specific sweetness level is ensured by careful selection of the timing of the fortification. According to the color, Port wines are classified as red (varying from deep purple to light gold), white (pale yellow to golden white), or *rosé* (pink related color) [4]. There are several styles of Port wines defined by the maturation method. It is worth noting that the selection of the fortification spirit is potentially relevant to each style, since the chemical composition of the distilled beverage will have an impact on several organoleptic characteristics of the final product, namely the color, taste, and aroma profile [3,29].

Ruby style is characterized by a deep red color, full body, fruity aromas, and rich tannins in the mouth. Ruby wines are commonly aged in large old wooden barrels called *Balseiro* (Figure 3), with the purpose of minimizing the contact between wine and oxygen, for a maximum of three years. This style of Port, in ascending order of quality, includes *Reserva*, Late Bottled Vintage (LBV), and Vintage. Vintage Port is produced from the grapes from a single harvest year, matured in wood for up to three years, and then submitted to a significant period, often many decades, of ageing in bottle. This style of Port wine presents a considerably different character compared to the wines matured uniquely in wood, and they are sold with the harvest date on the label [19].

Tawny Ports usually result from the blending of different batches of wine, aged in smaller vats or wood casks over different periods. This style is usually aged in wood for longer periods than the Ruby Ports. Contrarily to the Ruby style, the Tawny Ports undergo evolution in terms of color, which evolves from deep red to tawny, medium tawny or light tawny. The aroma is characterized by the notes of the dried fruits, spices, and wood. This style of Port includes *Reserva*, *Colheita* and Tawny with age indication. Reserve Tawny Ports must be aged in wood for a minimum of six years. Tawny with indication of age is commonly submitted to longer periods in wooden barrels with a volume of around 620–640 L, allowing a controlled exposure to oxygen and the transformation of the primary flavors of fruit into oxidation flavors, such as dried fruit, caramel, and nuts. *Colheita* Ports are Tawny Ports produced from a single vintage and aged in small wooden barrels for a minimum of seven years [19].

The production process for the White Ports differs in the use of decreased skin contact and lower fermentation temperatures, typically 17–18 °C, which permits achieving a fruitier aroma. White Ports are equally matured in old oak vats for a minimum of three years, prior to their commercialization. White Port aroma is defined by the duration of the ageing period, with young wines characterized by “crisp”, “fresh”, “acid”, “sweet”, “citrus fruit”, and “lemon balm” notes. Aged White Ports develop a “dry”, “woody”, “nutty”, and “incisive” flavor, due to the prolonged stage in wood and oxygen exposure [41].

## 4. Methodologies for the Characterization of Grape Spirits and Port Wines’ Volatile Components

Apart from the sampling and data processing and interpretation, the selection and optimization of the extraction methodology and the instrumental analysis are two fundamental steps in the construction of an analysis workflow, to provide high-quality data that may be useful to disclose information related to grape sprit and Port wine volatile composition and aroma characteristics, among others. The volatile profiles of the fortification grape spirits and Port wines have been studied using different extraction methodologies combined with chromatographic techniques, especially gas chromatography, as listed in Table 1. For each matrix, the references are listed in chronological order to assess trends over time in the techniques used.

As far as our research allowed, only four publications were identified for the determination of grape spirit volatile components [3,29,45,46], and 24 publications were reported for Port wine [3,6,8,9,11,12,13,14,15,16,17,21,23,24,26,27,46,47,48,49,50,51,52,53]. For untargeted analysis, liquid–liquid extraction (LLE) combined with gas chromatography coupled with mass spectrometry (GC-MS) has been mainly used for both matrices; however, in the last two decades, solid phase microextraction (SPME), a solvent-free technique, emerged as a suitable extraction approach to study these matrices. The targeted analyses, such as for carbonyl compound determination, were performed using derivatization followed by GC-MS [15,27,29,45], reverse-phase high-performance liquid chromatography coupled to a diode array detector (RP-HPLC-DAD) [46] and HPLC [16]. Sotolon detection is another example of target analysis performed on these matrices, and it was also determined using GC-MS [14] and RP-HPLC-DAD [51].

**Table 1:** Overview of the reported methodologies used for the untargeted or targeted analyses of grape spirits and Port wines’ volatile components.

Sample	Extraction Step			Chromatographic Analysis	Chemical Families/ Compounds Detected	Refs
	Sample Preparation	Extraction Methodology	Internal Standard			
Grape spirits	12.5 mL of grape spirit, diluted 1:4 with water	LLE - Hexane/diethyl ether (1:1, *v*/*v*) (4 + 4 + 2 mL)	4-Decanol (826 mg/L)	GC-ion trap MS Supelcowax 10 (60 m × 0.25 mm i.d., film thickness = 0.25 µm)	Alcohols, aldehydes, esters, phenols, terpenic compounds	[3]
Grape spirits	Without sample extraction, as grape spirit was directly injected		Methyl acetate (90 mg/L)	GC-FID CP Wax 52CB WCOT (60 m × 0.25 mm i.d., film thickness = 0.50 μm)	Acetaldehyde	[29]
Grape spirits	20 mL of grape spirit, diluted 1:1 with water	Derivatization + LLE - PFBOA as derivatization reagent (1 mL; 12 g/L; 1 h reaction; room temperature) - Saturation with 7 g of NaCl, followed by Ether-hexane (4 + 2 + 2 mL) extraction	Dodecanal (200 mg/L)	GC-ion trap MS Supelcowax 10 (60 m × 0.25 mm i.d., film thickness = 0.25 μm)	Aldehydes	[29]
Grape spirits	Without sample extraction, as grape spirit was directly injected		Methyl acetate (90 mg/L)	GC-FID CP Wax 52CB WCOT (60 m × 0.25 mm i.d., film thickness = 0.50 μm)	Acetaldehyde	[45]
Grape spirits	250 µL of grape spirit	Derivatization + HS-SPME - PFBHA as derivatization reagent (11.3 µL; 40 g/L; 10 min reaction; 38 °C) - PDMS/DVB - Vial: 20 mL - Extraction temperature: 38 °C - No salt addition - Stirring: 250 rpm - Extraction time: 30 min	Methyl acetate (90 mg/L)	GC-ion trap MS Fused silica capillary column * (30 m × 0.25 mm i.d., film thickness = 0.25 μm)	Aldehydes	[45]
Grape spirits	Derivatization + reduction methodology - 200 µL of grape spirit - Sulphur dioxide 6% (12 µL) - Sulphuric acid 70% (100 µL) - DNPH (1.5 mL) - Derivatization time of 15 min - Reduction time of 30 min, with sodium cyanoborohydride (200 mg)		*trans*-Stilbene (200 mg/L)	RP-HPLC-DAD Kinetex C18 column (100 mm × 4.6 mm, 2.6 μm diameter particles)	Acetaldehyde	[46]
Tawny, Ruby	700 mL of wine Nitrogen (50 mL/min) bubble through wine for 4 h	Porous Polymers Adsorption - Adsorbents: Tenax GC (60–80 mesh) or Porapak Q (50–80 mesh), both extractions were followed by a second Porapak Q trap - Thermal desorption 135 °C in Porapak Q and 250 °C in Tenax GC	-	GC-qMS Carbowax 20M (80 m × 0.76 mm glass) SF 96 (70 m × 0.76 mm glass) Carbowax 20M (25 m × 0.25 mm quartz)	Acids, alcohols, aldehydes, dioxanes, dioxolanes, esters, furans, lactones, ketones, norisoprenoids, phenols, sulphur compounds, terpenic compounds, other compounds	[8]
				GC-FID Glass Carbowax 20M coated SCOT (100 m × 0.76 mm) Quartz Carbowax 20M coated capillary (25 m × 0.25 mm)		
Tawny, Ruby	500 mL of wine	LLE - Continuous extraction with pentanemethylene chloride (2:1, 500 mL) for 8 h - Concentration using fractionating columns - Tawny Port (4.6 mL) was diluted in trichlorofluoromethane (5 mL) and extracted with 1,2-propanediol (2 × 3 mL + 1 × 2 mL)	-	GC-qMS Carbowax 20M (80 m × 0.76 mm glass) SF 96 (70 m × 0.76 mm glass) Carbowax 20M (25 m × 0.25 mm quartz)	Acids, alcohols, aldehydes, dioxanes, dioxolanes, esters, furans, lactones, ketones, norisoprenoids, phenols, sulphur compounds, terpenic compounds, other compounds	[8]
				GC-FID Glass Carbowax 20M coated SCOT (100 m × 0.76 mm) Quartz Carbowax 20M coated capillary (25 m × 0.25 mm)		
Port wines ^#^	50 mL of wine	LLE - Hexane/diethyl ether (1:1, *v*/*v*) (4 + 4 + 2 mL)	Isophorone (200 µg/L)	GC-ion trap MS Supelcowax 10 (60 m × 0.25 mm i.d., film thickness = 1 µm)	Acids, alcohols, aldehydes, esters, norisoprenoids, phenols, terpenic compounds, other compounds	[9]
Tawny, Ruby	50 mL of wine	LLE - Hexane/diethyl ether (1:1, *v*/*v*) (4 + 4 + 2 mL)	Isophorone (130 mg/L)	GC-ion trap MS Supelcowax 10 (60 m × 0.25 mm i.d., film thickness = 0.25 µm)	Acids, alcohols, esters, ketones, norisoprenoids, terpenic compounds	[12]
Port wine ^#^	50 mL table wine + 12.5 mL of grape spirits	LLE - Hexane/diethyl ether (1:1, *v*/*v*) (4 + 4 + 2 mL)	4-Decanol (826 mg/L)	GC-ion trap MS Supelcowax 10 (60 m × 0.25 mm i.d., film thickness = 0.25 µm)	Alcohols, aldehydes, esters, phenols, terpenic compounds	[3]
Port wine ^#^	50 mL of wine	LLE - Hexane/diethyl ether (1:1, *v*/*v*) (4 + 4 + 2 mL)	4-Decanol (826 mg/L)	GC-ion trap MS Supelcowax 10 (60 m × 0.25 mm i.d., film thickness = 0.25 µm)	1,3-Dimethoxybenzene	[11]
Port wine ^#^	50 mL of wine	LLE - Dichloromethane (5 + 5 mL)	Octan-3-ol (432.9 mg/L)	GC-qMS BP21 (50 m × 0.25 mm i.d., film thickness = 0.25 µm)	Dioxanes, dioxolanes, sulphur compounds	[13]
Tawny, Ruby	50 mL of wine	LLE - Dichloromethane (5 + 5 mL)	Ethyl (methylthio)acetate (0.050 mg/L)	GC-ion trap MS Stabilwax DA (60 m × 0.25 mm i.d., film thickness = 0.25 µm)	Sulphur compounds	[47]
Tawny, Ruby	50 mL of wine	LLE - Dichloromethane (5 + 5 mL)	Ethyl (methylthio)acetate (0.050 mg/L)	GC-FPD CP-Wax 58 (FFAP)-CB (50 m × 0.32 mm i.d., film thickness = 0.2 µm)	Sulphur compounds	[47]
Tawny	50 mL of wine	LLE - Dichloromethane (5 + 5 mL)	3-Octanol (432.9 mg/L)	GC-qMS BP21 (50 m × 0.25 mm i.d., film thickness = 0.25 µm)	5-(Hydroxymethyl)-2-furfural and sotolon	[48]
Tawny, Ruby	50 mL of wine	LLE - Dichloromethane (5 + 5 mL)	3-Octanol (466 mg/L)	GC-ion trap MS Stabilwax-DA (60 m × 0.25 mm i.d., film thickness = 0.25 µm)	Norisoprenoids	[6]
Tawny, Ruby	50 mL of wine	LLE - Dichloromethane (5 + 5 mL)	3-Octanol (466 mg/L)	GC-ion trap MS Stabilwax DA (60 m × 0.25 mm i.d., film thickness = 0.25 µm)	Sotolon	[14]
Port wine ^#^	40 mL of wine	SPE (clean up, column 1) + Derivatization + SPE (extraction, column 2) - LC18 column 1 g - Elution with water (5 mL) and acetonitrile (4 mL)	2,3-Hexanedione (25 mg/L)	HPLC Superspher 100 RP-18 (250 mm, 4 μm particle size) ^+^	Aldehydes, ketones	[16]
Tawny; Ruby	10 mL of wine	SPE - LiChrolut-EN cartridge (200 mg) - Elution with dichloromethane (2 mL)	2-Octanol (46.4 mg/L)	GC-ion trap MS DB-WAXETR (60 m × 0.25 mm i.d., film thickness = 0.25 µm) Precolumn Supelco (3m × 0.25 mm uncoated)	Aldehydes, sulphur compounds	[15]
White, Tawny, Ruby	100 mL of wine	SPE - LiChrolut-EN cartridge (200 mg) - Elution with dichloromethane (1.5 mL)	4-Hydroxy-4-methyl-2-pentanone (1500 mg/L)	PTV-GC–GC–MS (GC1: GC-FID; GC2: GC-ion trap MS, connected through a Deans switch) GC1: DB-WAX (30 m × 0.32 mm i.d., film thickness = 0.50 µm) GC2: FactorFour-VF5MS (30 m × 0.32 mm i.d., film thickness = 1µm)	Esters	[49]
Port wine	20 mL of wine	HS-SPME - DVB/CAR/PDMS (50/30 µm) - Vial: 40 mL - Extraction temperature: 35 °C - No salt addition - Stirring: 1300 rpm - Extraction time: 90 min	3-Octanol (47.7 mg/L)	GC-ion trap MS Stabilwax DA (60 m × 0.25 mm i.d., film thickness = 0.25 µm)	Norisoprenoids, terpenic compounds	[17]
Experimental Port wine	10 mL of experimental wine	HS-SPME - DVB/CAR/PDMS (50/30 µm) - Vial: 40 mL - Extraction temperature: 20 °C - Salt addition: 4 g - Stirring: 1300 rpm - Extraction temperature: 10 min (pre-equilibrium) + 60 min (extraction)	2-Octanol (0.10 mg/L)	GC-qMS Innowax (30 m × 0.25 mm i.d., film thickness = 0.5 µm)	Acids, alcohols, aldehydes, esters, ketones, norisoprenoids, phenols, sulphur compounds, terpenic compounds	[50]
Port wine	50 mL of wine	LLE - Dichloromethane (5 + 5 mL)	3-Octanol (427 mg/L)	GC-ion trap MS Stabilwax-DA (60 m × 0.25 mm i.d., film thickness = 0.25 µm)	Dioxanes, dioxolanes, furans, lactones	[53]
Port wine ^#^	50 mL of wine	LLE - Dichloromethane (5 + 5 mL)	3-Octanol (-)	GC-FID BP-21 (50 m × 0.25 mm i.d., film thickness = 0.25 μm)	Acids, alcohols, aldehydes, dioxanes, Dioxalanes, esters, furans, lactones	[21]
Port wine ^#^	50 mL of wine	LLE - Dichloromethane (5 + 5 mL)	3-Octanol (466 mg/L)	GC-ion trap MS Stabilwax-DA (60 m × 0.25 mm i.d., film thickness = 0.25 µm)	Alcohols, dioxanes, dioxolanes, esters, furans, lactones	[23]
Port wine ^#^	50 mL of wine	LLE - Dichloromethane (5 + 5 mL)	3-Octanol (427 mg/L)	GC-ion trap MS Stabilwax-DA (60 m × 0.25 mm i.d., film thickness = 0.25 µm)	Acids, aldehydes, esters, furans, lactones, phenols, other compounds	[24]
Port wine ^#^	9.9 mL of wine	HS-SPME - DVB/CAR/PDMS (50/30 µm) - Extraction temperature: 30 °C - No salt addition - Stirring: 500 rpm - Extraction time: 5 min (pre-equilibrium) + 5 min (extraction)	Trideuteriomethanol (30,000 mg/L) Pentan-1-ol (46,000 mg/L)	GC-qMS HP-INNOWa (30 m × 0.25 mm i.d., film thickness = 0.25 µm)	Alcohols, aldehydes, esters	[26]
White, Tawny, Ruby	2 mL of wine	Derivatization + HS-SPME - Prior derivatization with PFBHA as derivatization reagent (2.3 g/L) - PDMS/DVB (65 µm) - Vial: 5 mL - Extraction temperature: 32 °C - No salt addition - Stirring: 250 rpm - Extraction temperature: 10 min (pre-equilibrium) + 20 min (extraction)	p-Fluorobenzaldehyde (0.4 mg/L)	GC-TQ/MS BR-5 ms (30 m × 0.25 mm i.d., film thickness = 0.25μm)	Aldehydes, furans, lactones, ketones	[27]
Tawny, Ruby, White	10 mL of wine	LLE - Single-extraction with dichloromethane (6 mL) - Supernatant was submitted to a clean-up step with 3 mL of distilled water (3×) - Glycerol was used as a keeper	Veratric acid (200 mg/L)	RP-HPLC- DAD Kinetex C18 column (100 mm × 4.6 mm, 2.6 μm diameter particles)	Sotolon	[51]
Tawny, Ruby, White	Derivatization + reduction methodology - 200 µL of Port wine - Sulphur dioxide 6% (12 µL) - Sulphuric acid 70% (100 µL) - DNPH (1.5 mL) - Derivatization time of 15 min - Reduction time of 30 min, with sodium cyanoborohydride (200 mg)		Trans-stilbene (200 mg/L)	RP-HPLC-DAD Kinetex C18 column (100 mm × 4.6 mm, 2.6 μm diameter particles)	Aldehydes	[46]
Ruby	250 µL of wine	HS-SPME - DVB/CAR/PDMS (50/30 µm) - Vial: 20 mL - Extraction temperature: 45 °C - No salt addition - Stirring: 250 rpm - Extraction time: 5 min (pre-equilibrium) + 30 min (extraction)	-	GC-qMS Rxi-5Sil MS (30 m × 0.25 mm i.d., film thickness = 0.25 μm)	Aldehydes, esters, furans, lactones	[52]

For each matrix, the references are listed in chronological order to assess trends over time in the techniques used; ^#^ Port wine style not specified; + dicarbonyl and ketoacid compounds in samples were identified by comparison of the retention time in HPLC from commercially available standards and LCMSMS techniques; * columns used were not identified in the original paper. DNPH: 2,4-Dinitrophenylhydrazine; DVB/CAR/PDMS: divinylbenzene/carboxen/polydimethylsiloxane; FID: flame ionization detector; FPD: flame photometric detector; GC: gas chromatography; GC-qMS: gas chromatography coupled with quadrupole mass spectrometry; GC-TQ/MS: gas chromatography-triple quadrupole/mass spectrometry; HS-SPME: headspace—solid phase microextraction; IBCF: isobutyl chloroformate; LLE: liquid–liquid extraction; MS: mass spectrometry detector; PDMS/DVB: polydimethylsiloxane/divinylbenzene; q: quadrupole; PFBHA: O-(2,3,4,5,6-pentafluorobenzyl)hydroxylamine hydrochloride; PFBOA: [O-(2,3,4,5,6-pentafluorobenzyl) hydroxylamine; PTV—programmable temperature vaporization injector; RP-HPLC-DAD: reverse-phase high-performance liquid chromatography coupled to a diode array detector; SPE: solid-phase extraction.

LLE is a solvent extraction technique that has been commonly used for achieving exhaustive enrichment with the purpose of extracting volatile components from beverages, according to their partition coefficients between solvent and beverage. The procedure is laborious and, in addition to extraction, it also requires the concentration of the solutes by evaporation of the solvent. This stage can result in the loss of more volatile analytes, along with possible interferences derived from solvent impurities in the following chromatographic analysis. If the solvent evaporation is performed under vacuum and using a trap with liquid nitrogen, for more efficient condensation, it is possible to decrease the evaporation temperature, preventing and/or reducing the degradation of analytes or formation of artefacts [54]. Due to its intermediate polarity (dielectric constant ε = 9.1) and high volatility, dichloromethane has been widely used for the extraction of volatile compounds in these matrices (Table 1) [54,55]. However, as observed for other halogenated solvents, it represents some concerns for users and for the environment (carcinogenic, mutagenic, or toxic to reproduction substance—CMR) and, therefore, its use should be restricted and handled cautiously [55].

Further, there is a clear tendency to replace extraction with solvents by more environmentally friendly and solvent-free methodologies, such as SPME (Table 1). Additionally, in cases where solvents are used, there is a clear reduction of volume per extraction. These changes in the extraction methodologies also have an impact on the volume of sample used, which decreased from 700 mL for the first publication identified on volatile compounds of Port wine [8] to 250 µL using SPME extraction [52]. In fact, the extraction is one crucial step for the beverages’ volatile composition analysis, as it should provide a representative composition of the sample [56,57], including the highly volatile fraction, that can be lost in this step or may be co-eluted with the extraction solvent during the chromatographic analysis. SPME also has the advantage of allowing the determination of the more volatile components of the beverages that can potentially impact the aroma characteristics.

In fact, SPME is an alternative to perform the sampling and extraction/desorption of VOCs, which does not require expensive instrumentation, and, at the same time, fulfils the necessary requirements for implementation of green chemistry principles in analytical laboratories [58,59]. SPME is an extremely versatile technique, due to the vast number of fiber coatings available on the market that allow a broader extraction or a selective one, for targeting specifically the analytes of interest [60,61]. As observed in Table 1, divinylbenzene/carboxen/polydimethylsiloxane (DVB/CAR/PDMS) has been used for the extraction of VOCs from the headspace of grape spirits and Port wines [17,26], and polydimethylsiloxane/divinylbenzene (PDMS/DVB) has been applied in combination with derivatization [27] (Table 1). As the DVB/CAR/PDMS and PDMS/DVB fibers extract via an adsorptive-type mechanism, the analytes interact primarily with the surface of the sorbent coating instead of partitioning into the entire coating and, therefore, the sensitivity of these fibers depend on other factors such as the surface area and porosity of the material [59,62,63]. Due to the porosity properties of the DVB, the PDMS/DVB fiber may represent some concerns for the analyte displacement and has difficulty extracting analytes with low molecular weight, which explains why it is used to extract VOCs after derivatization (Table 1). The DVB/CAR/PDMS fiber, which combines three materials, was developed to overcome the limitations of CAR/PDMS in the desorption of higher molecular weight analytes and difficulty of PDMS/DVB in extracting analytes with low molecular weights. The DVB/CAR/PDMS coating contains two adsorbents that are layered to extend the molecular weight range of analytes extracted with one SPME fiber, and the combination with the PDMS confers it with a bipolar character, which explains the selection of this fiber in several studies [17,26,50,52], allowing the determination of a wide set of analytes, such as acids, alcohols, aldehydes, esters, terpenic compounds, volatile phenols, norisoprenoids, and furaldehydes.

Targeted determination of Port wines’ carbonyl compounds (combined with derivatization procedure) [15,16] and esters [49] was performed using solid phase extraction (SPE), associated with HPLC [16] and GC-MS [15,49]. Two solid sorbents, namely LiChrolut-EN [15,49] and C18 [16], were used. These two sorbents are widely used due to their properties, i.e., the performance of LiChrolut-EN cartridges for polar organic substances was explained based on its large specific surface, and C18 (octadecyl silica gel) selective extraction was controlled through hydrophobic interaction.

One-dimensional gas chromatography (1D-GC) has been used for the characterization of the volatile components of grape spirits and Port wine. Gas chromatographs equipped with flame ionization (FID) or mass spectrometry detectors with quadrupole (qMS) ion trap or triple quadrupole (TQ/MS) analyzers are usually employed. (Table 1). The FID detectors are significantly cheaper than the MS detectors, but the latter achieve lower detection and quantification limits. Moreover, commercial mass spectra databases are available for MS detectors, comprising an important tool for analyte identification, giving them a substantial advantage over GC-FID. Besides the library mass spectrum comparison and the possible co-injection of standards, there is another strategy to boost the putative identification of volatile components, namely the determination of linear retention indices (LRI). LRI is computed using the van Den Dool and Kratz equation after the injection of an *n*-alkanes series, thus improving the confidence in the analyte’s identification [64]. Moreover, currently GC-MS equipment features powerful software algorithms that facilitate data processing, and consequently data analysis [57,65,66].

The selection of the column is of utmost importance for the successful implementation of the chromatographic method [67,68]. Mainly polar stationary phases, such as polyethylene glycol (Stabilwax DA, Supelcowax 10, Carbowax 20M, DB-Wax and HP-INNOWax) and nitroterephthalic acid modified polyethylene glycol (BP-21), have been used for the volatile determination of fortification spirits and Port wines (Table 1), which may be justified, as polar or moderately polar analytes have been mainly determined.

In recent decades, several advances in the development of chromatographic equipment and software have allowed in-depth sample characterization, improving the limits of detection, chromatographic resolution, and reducing the time required for analysis and data processing [57]. Though the 1D-GC delivers a high-peak capacity, in the case of extremely complex matrixes, a single column is frequently not sufficient, resulting in overloaded chromatograms, due to the overlapping of compounds [65,69,70,71]. On the other hand, multidimensional gas chromatography, such as comprehensive two-dimensional chromatography (GC×GC), enables an in-depth characterization of complex samples due to the multiple sequential separations of a sample, using two columns with different stationary phases connected by an interface that allows one to preserve the individual analytes’ retention. Hence, comprehensive bidimensional gas chromatography has several advantages over conventional one-dimensional GC, including faster run times, lower limits of detection, superior resolution and peak capacity, and improved mass sensitivity and selectivity owing to the peak focusing on the modulator [57]. GC×GC has been successfully used for in-depth study of a wide range of alcoholic beverages, such as fortified Madeira wine [72,73], distilled spirit [74], table wine [75,76], sparkling wine [77], and beer [78,79]. Thus, it could prove to be very useful for a more in-depth study of the compounds associated with the aroma of grape spirit and Port wine.

## 5. Grape Spirits’ and Port Wines’ Volatile Components and Their Potential Impact on Aroma Properties

**Figure 4:**
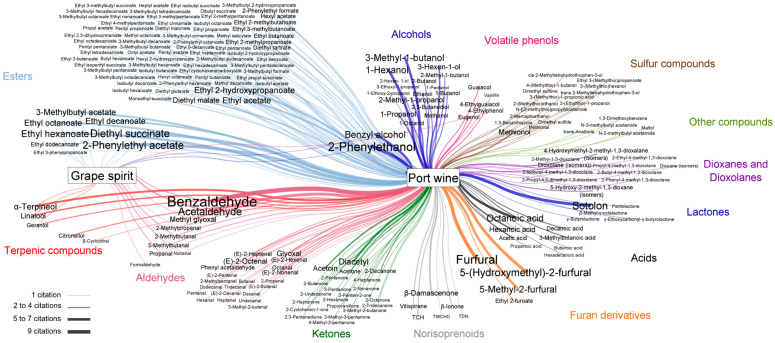
Visual representation of the volatile organic compounds reported in the literature for grape spirit (23 compounds) and Port wine (208 compounds)—for more details see Table S1, organized by chemical families. This figure was constructed using the software Cytoscape v3.9.1 (The Cytoscape Consortium, San Diego, CA, USA), in which nodes correspond to the type of beverage and the target nodes are the reported volatile components. Edge thickness is linked to the number of citations (between 1 and 9), as well as the size of the target nodes’ name. The compounds shared between both matrices are also visible (22 compounds).

Figure 4 is a visual representation of the volatile organic compounds reported in the literature for grape spirit used in the fortification of Port wine and for Port wine, including 23 and 208 compounds, respectively (for more details, see Table S1 [3,6,8,9,11,12,13,14,15,16,17,21,23,24,26,27,29,39,45,46,47,48,49,50,51,52,53]). The main two nodes of the figure correspond to the type of beverage, and the target nodes are the reported volatile components, which were organized by chemical families. A greater number of compounds have been identified in Port wine compared to grape spirit, but it is important to point out that there are also a higher number of publications on Port wine volatile composition (Table S1). Twenty-two compounds are reported in both matrices, listed in Table 2, which also includes the aroma descriptors and concentrations reported in both matrices. In fact, apart from formaldehyde, all 22 compounds detected in grape spirit were also detected in Port wine.

Grape spirit has reported in its composition 2 alcohols, 9 aldehydes, 8 esters, 1 phenol, and 3 terpenic compounds. Considering the Port wine, the reported volatile components include 8 acids, 18 alcohols, 29 aldehydes, 11 dioxanes and dioxalanes, 76 esters, 9 furaldehydes and lactones, 21 ketones, 6 norisoprenoids, 5 phenols, 15 sulphur compounds, 5 terpenic compounds, and 5 other compounds.

All 209 reported compounds systematized in Figure 4 cannot be found in only a single publication. Indeed, they are a result of the compilation of the reported research articles that studied different styles of Port wine and different grape spirit volatile compositions, which covered several analytical methodologies that were applied, thus leading to different compositions (Table S2 [3,11,12,14,15,47,52,80,81,82,83,84,85,86,87,88,89,90,91,92,93,94,95,96,97,98,99,100,101,102,103,104,105,106]). Benzaldehyde is the most cited, with a total of nine citations in Port wine [3,8,9,21,24,27,39,50,51]. Benzaldehyde has a bitter almond aroma descriptor [52], being associated with wine oxidation. Furthermore, it is interesting to observe that the aging of Port wine is a particular topic of interest. Indeed, several research papers focused on this theme, which contributed to the highest citations for furfural (7), 5-(hydroxymethyl)-2-furfural (6), sotolon (6), acetaldehyde (6), and 5-methyl-2-furfural (5). These compounds are reported as being formed by Maillard and/or oxidation reactions [21,23,24,52].

Esters constitute the major group of identified volatiles in both beverages, namely 8 and 76 reported volatile compounds in grape spirit and Port wine, respectively. Ethyl hexanoate (5 citations), 2-phenylethyl acetate (6 citations) and diethyl succinate (6 citations), are the most often referred esters (Figure 4). Moreover, there are other commonly reported volatile compounds that belong to the following chemical families: alcohols, such as 2-phenylethanol (7 citations), benzyl alcohol (5 citations), 3-methyl-1-butanol (5 citations), and 1-hexanol (5 citations); acids, namely octanoic acid (5 citations). These aforementioned volatile components are commonly known as being produced during yeast fermentation [107,108]. As a matter of fact, some of these components are reported with high concentrations in Port wine, for instance 2-phenylethanol (10,200–56,700 µg/L), 3-methyl-1-butanol (149,640–344,100 µg/L), acetaldehyde (1360–94,000 µg/L), and diethyl succinate (20–18,700 µg/L) (Table S1).

There are not enough data reported in the literature to directly relate the impact of the composition of a specific grape spirit on the composition of Port wine. However, an exploratory study performed by Rogerson and Freitas 2002 [3] showed a significant impact of the grape spirit on the aroma complexity of Port wine. Volatile compositions of both grape spirit and respective fortified Port wine were analyzed, unveiling an initial concentration increase (ca. 10 times) of fruity flavored ethyl esters in Port wines due to the fortification step, after which a chemical equilibrium was reached. Furthermore, Rogerson and Freitas 2002 [3] verified that several volatile compounds resulting from fortification had concentrations above their odor threshold in Port wines, and consequently were potentially contributing to Port wine aroma. These were ethyl hexanoate, ethyl octanoate and ethyl decanoate (fruity and tropical aromas); ethyl hydrocinnamate (fruity and balsamic aromas); and eugenol (spicy and clove aromas).

**Table 2:** Twenty-two volatile compounds that have been reported to be common in grape spirits and Port wine. The aroma descriptors and the concentration ranges in grape spirits and Port wine are also included.

Compound	Aroma Descriptor		Concentration Range in Grape Spirits (µg/L)		Concentration Range in Port Wine (µg/L)	
Phenylmethanol	Floral, sweet, disinfectant	[83,84]	100	[3]	85.3–2720	[3,50]
2-Phenylethanol	Rose, honey	[3,83,84,85,86]	1150	[3]	10,200–56,700	[3,9,50]
Acetaldehyde	Overripe apple	[84,87]	31,100–185,710	[29,45,46]	1360–94,000	[39,46,50]
2-Oxopropanal	Pungent, stinging	[106]	420–16,340	[29]	571–25,400	[16,27,39]
Propanal	Sharp and pungent	[106]	nd–220	[29,45]	4.1–403	[27]
2-Methylpropanal	Sharp, pungent	[106]	0.41–16.6	[29,45]	24–1087	[15,27]
2-Methylbutanal	Powerful, choking	[106]	0.19–5690	[29,45]	17–806	[15,27]
3-Methylbutanal	Choking, powerful, acrid, pungent, apple-like	[106]	9.07–4140	[29,45]	20–2246	[15,27]
Benzaldehyde	Smokey, nutty, almond	[3,83,84,85]	20–690	[3,29]	0.79–837	[3,9,27,39,50,52]
Nonanal	Fatty	[106]	0.211–33.5	[45]	1.2–3.1	[27]
3-Methylbutyl acetate	Banana, fruity, sweet	[3,83,85,86,87]	434	[3]	330–1269	[3,39,50]
Diethyl succinate	Fruity, melon, yeasty	[3,83,84,85,86,87]	6500	[3]	20–18,700	[3,39,50]
Ethyl hexanoate	Fruity, green apple, banana, brandy, wine-like	[3,83,84,85,86,87]	827	[3]	109–1097	[3,39,50]
2-Phenylethyl acetate	Flowery, honey	[3,83,84,85,86]	55.4	[3]	11.6–1179	[3,9,50]
Ethyl octanoate	Sweet, floral, fruity, banana, pear, brandy	[3,83,84,85,86,87]	3210	[3]	56–3180	[3,39,50]
Ethyl 3-phenylpropanoate	Floral, sweet, fruity	[3,83]	2.2	[3]	3.5–6.7	[3]
Ethyl decanoate	Brandy, fruity, grape, chemical	[3,83,85,86,87]	4600	[3]	60.9–4490	[3,50]
Ethyl dodecanoate	Sweet, floral, fruity, cream	[3,83,86]	441	[3]	491–892	[3]
Eugenol	Cinnamon, clove, honey	[84,87,88]	1.8	[3]	5.2–10.3	[3,9]
Linalool	Citrus, floral, sweet, lavender	[83,85,87]	20.3	[3]	1.22–61.0	[3,9,50]
Geraniol	Floral, sweet	[3]	12.7	[3]	10.5–61.4	[3,9]
α-Terpineol	Lilac, floral, sweet	[83,84,85,86]	20.1	[3]	10.1–58.2	[3,9]

nd: not detected.

**Figure 5:**
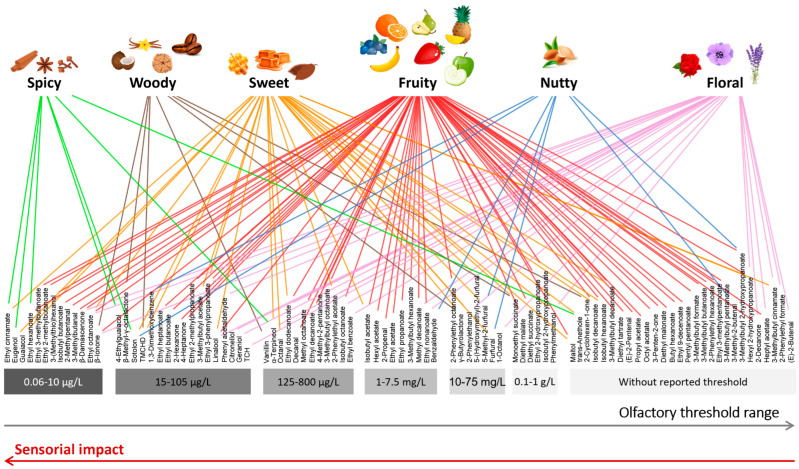
Organization of the VOCs using two main types of information: One side with the six valuable classes of aromas in Port wines (spicy, woody, sweet, fruity, nutty and floral notes), and the other side with the range of olfactory thresholds (Table S2). Each volatile component was distributed according to its aroma descriptor(s) and the respective olfactory threshold reported on Port wine (limited data are available for this matrix) or hydroalcoholic matrices, ranging from a few µg/L to g/L. VOCs considered potential contributors to these aromas, even without reported olfactory thresholds in ethanol/water matrix, were also included.

According to the IVDP, there are specific aromas that are valued in the various Port wine styles [1]. For instance, young Port wines have characteristic fruity and floral aromas, and these aromas may evolve and change through ageing in oak barrels, which then provide distinct and unique complex aromas to aged Port wine, such as honey, dry fruit, spicy and nutty [1,19]. Thus, from all of the volatile compounds reported in Port wines (Tables S1 and S2), a selection was performed, taking into consideration the ones with the aromas related to the six classes of aromas that are appreciated in the various Port wine styles: spicy, woody, sweet, fruity, nutty and floral [1]. Thus, Figure 5 was constructed using two main types of information: one side with the six classes of valued aromas in Port wines, and on the other side the range of the olfactory thresholds (based on data reported in Table S2). Each volatile compound was distributed according to its aroma descriptor(s) and the respective reported olfactory threshold in Port wine (limited data are available for this matrix) or in hydroalcoholic matrices, ranging from few µg/L to g/L. This organization allowed us to visualize which volatile compounds may be responsible for specific aromas and their potential sensorial impact, since the lower the olfactory threshold, the higher the potential sensorial impact that volatile compound may have in Port wine. Additionally, some of the compounds that do not have reported olfactory thresholds were also included in Figure 5. It is important to point out that the matrix in which the olfactory threshold is determined has an impact on the obtained value, for instance, the ethanol and sugar content will influence the odor perception. Only three research papers have reported olfactory thresholds in Port wines [14,52,89]; therefore, it was necessary to expand the search for olfactory thresholds. Only the hydroalcoholic matrices, in which ethanol content varied between 9 and 14% *v*/*v*, were selected from the reported literature (Table S2). Even though these ethanol concentrations are lower than the ones in Port wines (18–22 and 16.5% *v*/*v* for red and white wines, respectively [1,39]), they were the closest matching matrices, and only those were considered in Figure 5.

Each aroma class (spicy, woody, sweet, fruity, nutty, and floral) included several other reported aromas associated with those; in this case, spicy aroma notes incorporated cinnamon, anise, clove; woody aroma notes also comprised vanilla, coconut, coffee, and toasted; sweet aroma notes covered honey, caramel, and cocoa; fruity aroma notes contained tree fruit, tropical fruit, berry fruit, and citrus; nutty aroma notes encompassed also almond; and floral aroma notes involved rose, lavender, hyacinth, lilac, and orchid.

Fruity is the aroma class with the highest number of volatile compounds, and their sensorial impact varies significantly, ranging from those with the lowest (0.06–10 µg/L) to the highest (0.1–1 g/L) reported olfactory thresholds (Table S2), e.g., 5 µg/L for ethyl hexanoate (banana and green apple aroma), 203 µg/L for methyl octanoate (orange aroma), or 0.76 g/L for diethyl malate (over-ripe peach, prune aroma) Indeed, most of the fruity aromas arise from esters. Moreover, some ketones present fruity aroma notes, e.g., 2-hexanone (fruity, fungal, meaty, buttery aroma, 24 µg/L).

Several chemical families may contribute to floral aromas; for instance, esters, alcohols, terpenic compounds, and norisoprenoids, which have olfactory thresholds between 15 and 800 µg/L, e.g., citronellol (rose aroma, 100 µg/L), linalool (floral, rose, lavender aroma, 15 µg/L), or 2-phenylethyl acetate (floral and rose aroma, 250 µg/L). Norisoprenoids such as β-damascenone (cooked apple, floral, and honey aroma, 0.1 µg/L) and β-ionone (violet, woody, raspberry, rose aroma, 0.1 µg/L) have the lowest reported olfactory thresholds, therefore, the highest sensorial floral perception. On the contrary, alcohols have the highest reported olfactory thresholds, such as 2-phenylethanol (floral, rose and honey aroma, 12 mg/L) and 1-octanol (jasmine, rose, waxy, and soapy aroma, 10 mg/L).

Esters, alcohols, and ketones that contribute to fruity and floral aromas may arise from yeast fermentation, e.g., from carbohydrate metabolism, from lipid oxidation, and/or from amino acid metabolism [39,108]. Furthermore, terpenic compounds are varietal components once they are secondary metabolites from grapes, but they also can be biotransformed along with yeast fermentation [109,110]. Norisoprenoids are produced from the breakdown of carotenoids (present in grapes) [109].

Sweet aroma notes can be associated with furan derivatives and lactones. Some esters, terpenic compounds, norisoprenoids, and volatile phenols also have been reported to have sweet aroma descriptors. Their sensorial impact is quite diverse due to the wide range of reported olfactory thresholds that are associated with these compounds (also including compounds with no reported olfactory threshold), for instance: 10.3 µg/L for guaiacol (sweet, smoke, medicinal aroma); 250 µg/L for α-terpineol (lilac, floral, sweet, and anise aroma), 16 mg/L for 5-methyl-2-furfural (almond, caramel, and burnt sugar aroma), or 1 g/L for monoethyl succinate (caramel and coffee aroma).

Considering the spicy aromas (including cinnamon, anise, clove aroma), their sensorial impact is quite high, once the compounds that contribute with these aromas have low reported olfactory thresholds (most of them between 1.1 to 41 µg/L). Few compounds have been reported to have olfactory spicy notes, e.g., ethyl cinnamate (cinnamon, balsamic, and honey aroma, 1.1 µg/L), eugenol (cinnamon, clove and honey aroma, 5 µg/L), or 4-ethylguaicol (spice, clove, toasted bread, and smoky aroma, 41 µg/L).

Woody aromas, including vanilla, coconut, coffee, and toasted, are characteristic of volatile phenols and norisoprenoids. Indeed, volatile compounds such as β-ionone (0.1 µg/L), β-methyl-γ-octalactone (oaky, coconut and vanilla aroma, 54.5 µg/L), and vanillin (vanilla aroma, 200 µg/L) may have an important sensorial impact for woody aromas, while γ-butyrolactone (caramel, sweet, and coconut aroma, 75 mg/L) and monoethyl succinate (1 g/L) require higher concentrations to be sensorially perceived.

Seven volatile compounds from different chemical families may contribute to nutty aromas in Port wine, with sensorial impact differing according to the olfactory threshold. Indeed, sotolon (nutty, spicy, perceived aged aromas) and 1,3-dimethoxybenzene (medicinal and hazelnut aroma) have the lowest reported olfactory thresholds, namely 19 and 47 µg/L, respectively, thus their sensorial impact should be higher than the one expected for benzaldehyde (almond, nutty, and smoky aroma), furfural (bread, almond, and sweet aroma) or 5-methyl-2-furfural, whose reported olfactory thresholds are 5, 15 and 16 mg/L, respectively.

Most of the volatile components that contribute to sweet, spicy, woody and nutty aromas to Port wine are ageing-related compounds, belonging to the following chemical families: volatile phenols, lactones, furan derivatives, and/or norisoprenoids. Their concentrations and consequently their sensorial impacts are dependent upon the ageing conditions, particularly the length of ageing and wood type. Compounds such as eugenol might be the result of the thermal degradation of lignin, being posteriorly extracted from the wood during oak barrel ageing [111]. Wood-extracted compounds also include other volatile phenols, such as phenol, vanillin, 4-ethylphenol, and 4-ethylguaiacol, and lactones, particularly β-methyl-Υ-octalactone [39]. Moreover, furan derivatives, oxygen-containing heterocyclic compounds, can originate from carbohydrate degradation, non-enzymatic browning reactions, and aldehyde polymerization, with their content being linearly correlated with the duration of the wood maturation period [39,108].

## 6. Global Outlook and Future Challenges

Port wine is a key product for the national economy of Portugal and a symbolic asset representing the country worldwide. Port wine is a fortified wine based upon a long tradition with expert blending employed for style, creating defined character, and producing the quality factor that makes Port wine highly prized. The fermentation stoppage, by adding a non-highly rectified grape spirit to achieve a final alcoholic content of around 19% (*v*/*v*), represents a distinctive and crucial step for Port wine characteristics [4,5]. In fact, apart from increasing the ethanol content, the grape spirit was also shown to influence the final organoleptic characteristics of Port wine, namely the color, taste, and aroma, by contributing with several VOCs that reach or surpass the sensory perception limits [3,29]. Likewise, the impact of fortification on sensorial perception can be explored at various levels; a recent study has shown the functionality of ethanol on VOCs—α-amylase interactions—and on the release of VOCs based on their molecular hydrodynamics [112].

As far as we know, this review contains the most extensive database on the volatile composition of grape spirit and Port wine, corresponding to 23 and 208 compounds, respectively. This information is scattered in some publications, and comparative analyses of different types of alcoholic beverages were performed, in which Port wine was also included, but no specific information about style, age, among others, were reported. Therefore, it was not possible to carry out an in-depth discussion and possible stratification of information by Port wine style. In fact, understanding the components of Port wine aromas, and of the respective grape spirits, has become more important than ever with the transformation of food systems, lifestyles of consumers and the increased demands for innovation in the food industry, even in the traditional ones. To go further into that direction, it is crucial to characterize a wide set of Port wines and grape spirits, well defined according to style, year, winemaking process, harvest, producer, etc., using advanced analytical tools. To advance in this challenging topic, it is crucial to apply high-throughput techniques, such as GC×GC-ToFMS, which seems to be a powerful technique for the analytical coverage of the chemical data on volatile compounds, or even used in combination with olfactometry and/or advanced artificial intelligence techniques. These chemical data seem to be useful in constructing predictive models to provide insights into the human perception of odorants, to predict odor from molecular structure, and to decode the relationships between smell, olfactory receptors and VOCs, among others [113,114,115].

Due to the huge significance of human olfaction in several fields, namely in understanding consumer preferences and consumption, measuring and chemically revealing the smells represent cutting-edge research, whose importance is expected to increase in the future. Though it is still unclear how odorants are recognized by odorant receptors, our sense of smell enables us to travel through a vast space of chemically diverse odorant molecules that in a combinatorial way activate approximately 400 odorant G protein-coupled receptors encoded in the human genome [116]. Thus, as deeper information is obtained about this broad range of odorant molecules of grape spirits and respective Port wines, it may be used as a support tool in the decision-making process, namely in the selection of grape spirits for fortification. This objective information on the chemical aromas of spirits and Port wines may be used by producers to innovate and produce wines with differentiating characteristics, in line with current market trends that seek genuine products that provide new sensory experiences.

## Data Availability

All related data and methods are presented in this paper. Additional inquiries should be addressed to the corresponding author.

## Supplementary Materials

The following supporting information can be downloaded at: https://www.mdpi.com/article/10.3390/foods12122432/s1, Table S1: Volatile compounds reported in the literature for grape spirit (aguardente) and Port wine, organized by chemical family; Table S2: List of volatile compounds reported in the literature in grape spirits and Port wine, with the respective aroma descriptors, olfactory threshold (OT) (µg/L) and respective matrix where they were determined.
